# Surface display of ACC deaminase on endophytic *Enterobacteriaceae* strains to increase saline resistance of host rice sprouts by regulating plant ethylene synthesis

**DOI:** 10.1186/s12934-017-0831-5

**Published:** 2017-11-28

**Authors:** Yupei Liu, Lixiang Cao, Hongming Tan, Renduo Zhang

**Affiliations:** 1 School of Environmental Science and Engineering, Guangdong Provincial Key Laboratory of Environmental Pollution Control and Remediation Technology, Sun Yat-sen University, Guangzhou, 510006 China; 2 School of Life Sciences, Guangdong Provincial Key Laboratory for Climate Change and Natural Disaster Studies, Sun Yat-sen University, Guangzhou, 510275 China

**Keywords:** ACC deaminase, Engineered strains, Endophytes, Rice, Saline stress

## Abstract

**Background:**

Most endophytic bacteria in consortia, which provide robust and broad metabolic capacity, are attractive for applications in plant metabolic engineering. The aim of this study was to investigate the effects of engineered endophytic bacterial strains on rice sprout ethylene level and growth under saline stress. A protocol was developed to synthesize engineered strains by expressing bacterial 1-aminocyclopropane-1-carboxylate (ACC) deaminase gene on cells of endophytic *Enterobacter* sp. E5 and *Kosakonia* sp. S1 (denoted as E5P and S1P, respectively).

**Results:**

Results showed that ACC deaminase activities of the engineered strains E5P and S1P were significantly higher than those of the wild strains E5 and S1. About 32–41% deaminase was expressed on the surface of the engineered strains. Compared with the controls without inoculation, inoculation with the wild and engineered strains increased the deaminase activities of sprouts. Inoculation with the engineered strains increased 15–21% more deaminase activities of sprouts than with the wild strains, and reduced the ethylene concentrations of sprouts more significantly than with wild strains (*P* < 0.05). Inoculation with the wild and engineered strains promoted the growth of sprouts, while the promoting effects were more profound with the engineered strains than with the wild strains. The engineered strains improved saline resistance of sprouts under salt concentrations from 10 to 25 g L^−1^. The engineered strains promoted longer roots and shoots than the wild strains under the salt stresses, indicating that the ACC deaminases on the endophytic bacterial cells could result in plant-produced ACC degradation and inhibit plant ethylene formation.

**Conclusions:**

The protocols of expressing enzymes on endophytic bacterial cells showed greater potentials than those of plant over-expressed enzymes to increase the efficiency of plant metabolic pathways.

**Electronic supplementary material:**

The online version of this article (10.1186/s12934-017-0831-5) contains supplementary material, which is available to authorized users.

## Background

Plant secondary metabolism has multiple functions throughout the plant life cycle, such as generation of secondary metabolites. Many plant secondary metabolites are used for production of medicines, dyes, insecticides, spices, and fragrances [1]. The secondary metabolism is an interesting target for plant genetic engineering. In the past 20 years, although some research activities have been conducted in the field, the plant secondary metabolic pathways at the level of biosynthetic intermediates and enzymes are still poorly understood [1, 2]. The consecutive enzymes are probably organized into macromolecular complexes that are associated with the endomembrane system [3]. In the plant metabolic engineering, results of over-expression of a few judiciously chosen enzymes are often disappointing due to lack of understanding of metabolic regulation and possible toxicity of end products [4]. Therefore, approaches beyond enzyme over-expression should be developed.

A wide diversity of microorganisms live both inside and outside plant tissues and these microbiota are involved in plant nutrition and plant resistance to biotic and abiotic stresses [5]. Plants are no longer viewed as autonomous entities, but as the host to include all of its symbiotic microbes and hologenome consisting of the nuclear genome, organelles, and microbiome [6, 7]. As an integral part of the plant hologenome, the plant microbiome can be selected with the plant genome to develop next-generation plant breeding approach [8]. Most of natural microbes exist in consortia to provide robust and broad metabolic capacity and these traits are attractive for applications in the plant metabolic engineering [9]. Synthetic metabolons, in which enzymes are scaffolded to synthetic proteins, have been expressed in microorganisms. It has been shown that scaffolded enzymes are more effective than free enzymes for metabolic engineering [4]. Ethylene is an efficient plant growth regulator at a very low concentration (e.g., 0.05 μL L^−1^) under stress conditions, such as salinity and drought stresses. Ethylene can be synthesized from S-adenosyl methionine by 1-aminocyclopropane-1-carboxylate (ACC) synthase and ACC oxidase in plants [10]. Therefore, ACC is an important precursor of ethylene in plants. Bacterial ACC deaminases promote plant growth by sequestering and cleaving plant produced ACC, and thereby reducing the ethylene level in the plant [10]. The expression of ACC deaminases by symbiotic bacteria can alleviate the ethylene-mediated negative impact on plants [11, 12]. Thus the ACC deaminase can be used as a good model to study the effects of symbiotic bacterial enzymes on the outcome of metabolic engineering in plants.

**Fig. 1:**
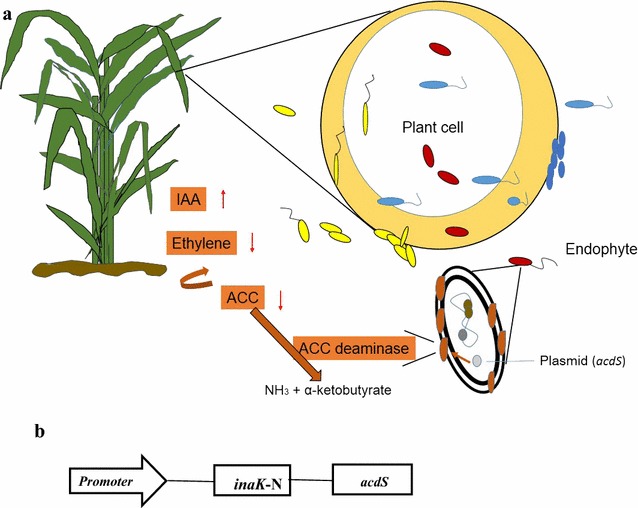
**a** A schematic graph to show endophyte surface display ACC (1-aminocyclopropane-1-carboxylate) deaminase and stimulated IAA (indole-3-acetic acid) to facilitate plant growth. **b** Gene map of recombinant plasmid. Plasmid pUC57 was used as a parent vector for constructing the recombinant plasmid

Although ACC is a plant product, ACC deaminase is not a secreted enzyme and within the cytoplasm of endophytes. Therefore, a protocol was developed to synthesize engineered strains by expressing bacterial ACC deaminase gene on cell surface of rice endophytes (Fig. 1a). The hypothesis in this study was that introducing engineered endophytic bacteria should improve the metabolite synthesis of plants, such as reduction of ethylene levels and enhancement of saline resistance. To test this hypothesis, two engineered endophytic *Enterobacteriaceae* strains were produced by expressing ACC deaminase (*acdS*) on bacterial cells and the engineered strains were inoculated into host rice sprouts to investigate ethylene levels and saline resistance of rice sprouts under saline stress.

## Results

### Isolation of endophytic bacteria and construction of strains displaying ACC deaminase

After incubated at 26 °C for 4 days, pink colonies grown on the sprout samples were picked and further purified on the EMB medium with 1.5% agar. The purified strains (wild strains) were identified as *Enterobacter* sp. E5 and *Kosakonia* sp. S1 with the 16S rDNA gene sequence analysis.

The synthesized fragments from 2 to 3 kb were eluted from agarose gel and ligated into pUC57 plasmid. Clones on LB plates were screened for ACC deaminase activity and the selected clones were sequenced by Invitrogen (Guangzhou, China). The clones were convinced using double digestion and sequencing of plasmids. The transformed E5 and S1 cells were designated as engineered strains of E5P and S1P, respectively.

### Measurement of enzyme activities

Activities of ACC deaminase of the engineered strains were significantly higher than those of the wild strains with and without disruption of toluene. The ACC deaminase activities of the wild strains were not detected when the cells were not disrupted by toluene, thus the bacterial ACC deaminase was intercellular (Table 1). The ACC deaminase was located on the surface of E5P and S1P because higher ACC deaminase was displayed on the engineered strains than on the wild strains. The ACC deaminase activities of the engineered strains increased significantly when the strains were disrupted by toluene (Table 1). About 41 and 32% deaminase was expressed on the surface of E5P and S1P, respectively.

**Table 1:** The ACC deaminase activities (concentration of α-ketobutyrate, mM) of the wild strains of endophytic *Enterobacter* sp. E5 and *Kosakonia* sp. S1, and their *acdS* gene surface expressed strain (i.e., the engineered strains E5P and S1P, respectively) with and without disruption of toluene

	E5	E5P	S1	S1P
With toluene	0.23 ± 0.03^c^	5.75 ± 1.16^b^	0.38 ± 0.06^c^	7.29 ± 0.65^a^
Without toluene	0.09 ± 0.02^b^	2.37 ± 0.38^a^	0.16 ± 0.08^b^	2.33 ± 0.35^a^

The results were represented with values of the mean ± standard deviation of three replicates. Within each row, values followed by the different superscripts are significantly different (*P* < 0.05)

### ACC deaminase activity and ethylene content in rice sprouts

The ACC deaminase activities of rice sprouts were further measured to determine whether the engineered endophytic strains displayed deaminase *in planta*. Compared with the controls, inoculation with the wild and engineered strains obviously increased the deaminase activities of sprouts. Sprouts inoculated with the engineered strains increased deaminase activities by 15 and 21% than those with the wild strains (Table 2). According to the deaminase activities of sprouts, the ethylene concentrations of sprouts declined with the inoculation of the engineered strains (Table 2). Therefore, the ACC deaminase expressed on the cells of E5 and S1 could reduce the ethylene concentrations of rice sprouts.

**Table 2:** The ACC deaminase activity (concentration of α-ketobutyrate, mM) and ethylene concentrations (nmol L^−1^) of sprouts for rice seeds treated with sterile water (the control), the wild strains (i.e., endophytic *Enterobacter* sp. E5 and *Kosakonia* sp. S1), and their *acdS* gene surface expressed strain (i.e., the engineered strains E5P and S1P, respectively)

	Control	S1	S1P	E5	E5P
Concentration of α-ketobutyrate (mM)	0.72 ± 0.02	0.81 ± 0.03*	0.98 ± 0.05*	1.00 ± 0.01*	1.15 ± 0.01*
Concentration of ethylene (nmol L^−1^)	88.51 ± 2.32	78.84 ± 6.73	66.94 ± 2.07*	67.56 ± 2.05*	61.36 ± 1.70*

The results were represented with values of the mean ± standard deviation of three replicates

* Values are significantly different from the control (*P* < 0.05)

### Effects of the endophytic bacteria on root lengths and weights

**Fig. 2:**
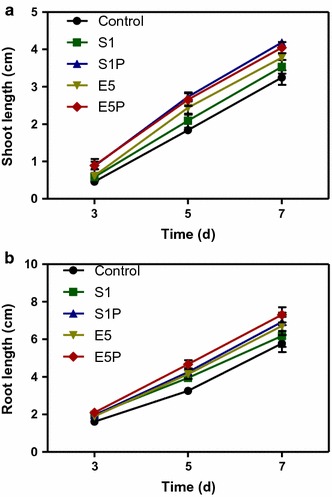
**a** Shoot length and **b** root length for rice seeds treated with sterile water (the control), the wild strains (i.e., endophytic *Enterobacter* sp. E5 and *Kosakonia* sp. S1), and their *acdS* gene surface expressed strain (i.e., the engineered strains E5P and S1P, respectively). Error bars indicate standard errors (*n* = 100 seeds divided in five plates)

The growth of sprouts was promoted by the inoculation with rice endophytes E5 and S1. Shoot and root lengths of sprouts inoculated with E5 and S1 increased significantly compared with the controls without inoculation during the period 3–7 days (Fig. 2a, b). The engineered strains E5P and S1P showed higher growth promoting effects on sprouts than the wild strains E5 and S1, respectively (Fig. 2). The biomass (fresh and dry weights) of shoots and roots increased significantly with inoculation of the engineered strains. However, inoculation with the wild strains did not increase the sprout biomass significantly (Table 3).

**Table 3:** Fresh weights of shoot and root (g), dry weight of shoot and root (mg) for one plate of 20 seeds treated with sterile water (the control), the wild strains (i.e., endophytic *Enterobacter* sp. E5 and *Kosakonia* sp. S1), and their *acdS* gene surface expressed strain (i.e., the engineered strains E5P and S1P, respectively)

	Control	S1	S1P	E5	E5P
Fresh weight of shoot (g)	0.25 ± 0.02^d^	0.29 ± 0.03^c^	0.35 ± 0.03^a^	0.30 ± 0.02^bc^	0.33 ± 0.03^ab^
Dry weight shoot (mg)	25.60 ± 3.05^c^	29.94 ± 4.62^c^	39.94 ± 6.11^a^	32.02 ± 2.57^bc^	37.72 ± 7.49^ab^
Fresh weight of root (g)	0.22 ± 0.03^c^	0.26 ± 0.03^bc^	0.37 ± 0.06^a^	0.30 ± 0.02^b^	0.37 ± 0.03^a^
Dry weight of root (mg)	25.74 ± 7.91^c^	32.44 ± 6.45^bc^	50.30 ± 6.92^a^	36.94 ± 2.30^b^	51.34 ± 3.22^a^

The results were represented with values of the mean ± standard deviation of five replicates. Within each row, values followed by the different superscripts are significantly different (*P* < 0.05)

### Effects of the engineered strains on rice under saline stress

**Fig. 3:**
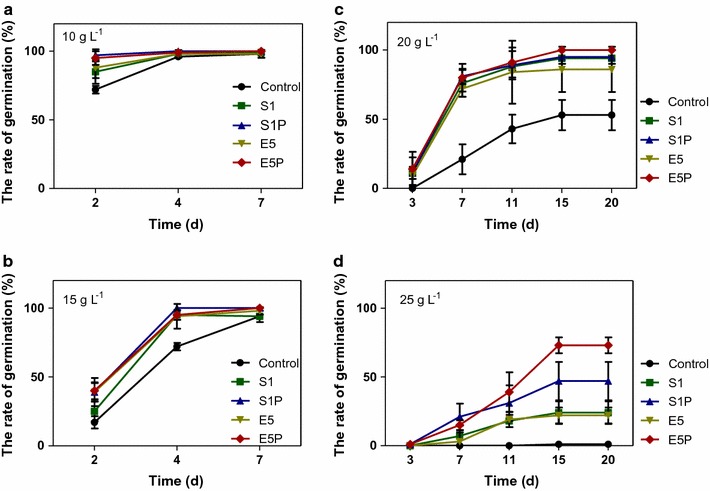
Germination rates (%) of rice seeds treated with sterile water (the control), the wild strains (i.e., endophytic *Enterobacter* sp. E5 and *Kosakonia* sp. S1), and their *acdS* gene surface expressed strain (i.e., the engineered strains E5P and S1P, respectively) under the saline concentrations of **a** 10 g L^−1^, **b** 15 g L^−1^, **c** 20 g L^−1^, and **d** 25 g L^−1^. Error bars indicate standard errors (*n* = 100 seeds divided in five plates)

Under the saline stresses, the germination rates of rice seeds were retarded (Fig. 3). With the salt concentrations increased to 25 g L^−1^, germination rates of the control seeds were completely inhibited. The inoculation with E5P and S1P into rice seeds increased saline resistance of sprouts under the salt concentrations increased from 10 to 25 g L^−1^ (Additional file 1: Figures S1, S2, S3, and S4). With the salt concentrations from 10 to 25 g L^−1^, inoculation with the engineered strains improved germination rates more significantly than inoculation of the wild strains. Under the concentration of 25 g L^−1^, germination rates of the seeds inoculated with E5P were significantly higher than those with S1P (Additional file 1: Figure S4). The differences between germination rates of seeds inoculated with E5 and S1 were not significantly (Fig. 3).

**Fig. 4:**
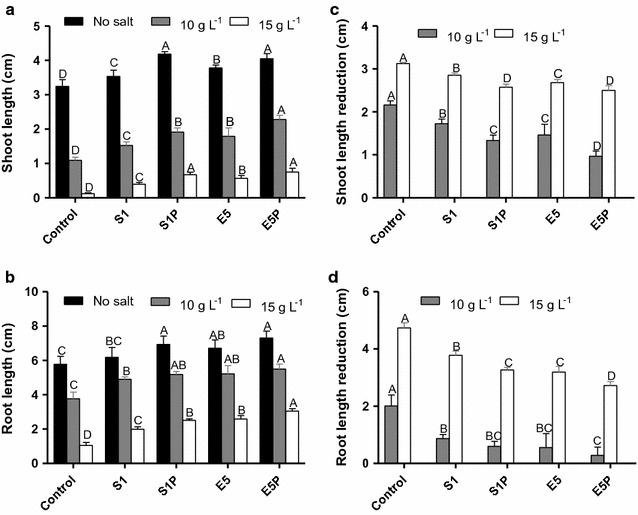
**a** Shoot length under the saline concentrations of 0, 10, and 15 g L^−1^, **b** root length under the saline concentrations of 0, 10, and 15 g L^−1^, **c** shoot length reduction under the saline concentrations of 10 and 15 g L^−1^, and **d** root length reduction under the saline concentrations of 10 and 15 g L^−1^ for rice seeds treated with sterile water (the control), the wild strains (i.e., endophytic *Enterobacter* sp. E5 and *Kosakonia* sp. S1), and their *acdS* gene surface expressed strain (i.e., the engineered strains E5P and S1P, respectively). Error bars indicate standard errors (*n* = 100 seeds divided in five plates). Capital letters above bars indicate that values are significantly different between the treatments for each salt concentration (*P* < 0.05)

Although the germination rates of rice seeds were not influenced by the saline stress of 10 and 15 g L^−1^, the growth of rice sprouts were significantly retarded (Fig. 4). Nevertheless, inoculation of E5 and S1 improved sprout growth under the salt concentrations of 0, 10, and 15 g L^−1^. The engineered strains E5P and S1P showed better effects on sprout growth than E5 and S1, respectively (Fig. 4). Shoot and root lengths of sprouts inoculated with E5P were longer than those with S1P, E5, and S1. While the saline stresses of 10, 15, 20, and 25 g L^−1^ reduced both root and shoot lengths of the control treatment, the sprouts inoculated with the wild and engineered strains increased tolerance to saline stress. Furthermore, expressing the *acdS* gene on E5 could obtain longer roots and shoots than on S1 under the salt stresses (Fig. 4a, b).

**Fig. 5:**
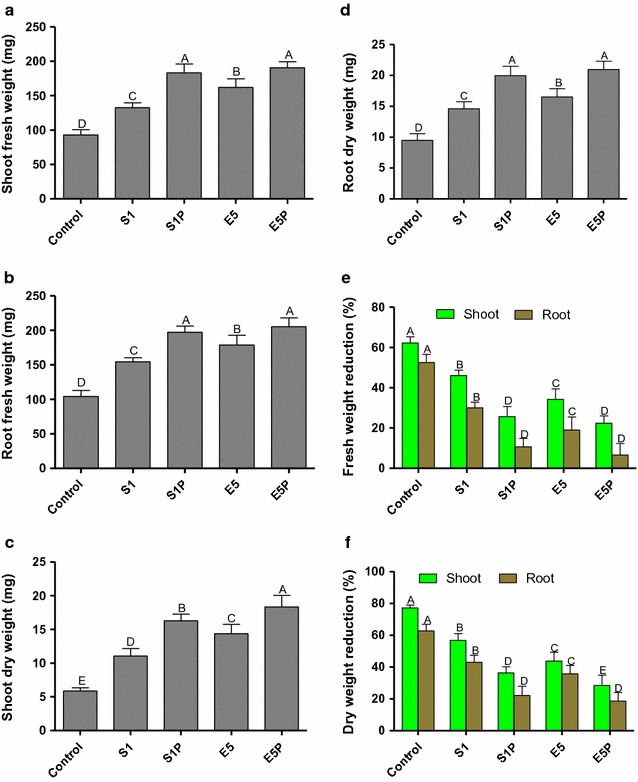
**a** shoot fresh weight, **b** root fresh weight, **c** shoot dry weight, **d** root dry weight, **e** fresh weight reduction, and **f** dry weight reduction for rice seeds treated with sterile water (the control), the wild strains (i.e., endophytic *Enterobacter* sp. E5 and *Kosakonia* sp. S1), and their *acdS* gene surface expressed strain (i.e., the engineered strains E5P and S1P, respectively) under the saline concentration of 10 g L^−1^. Error bars indicate standard errors (*n* = 100 seeds divided in five plates). Capital letters above bars indicate that values are significantly different between the treatments (*P* < 0.05)

Under the saline stresses of 10, 15, 20, and 25 g L^−1^, the growth of sprouts inoculated with E5 and S1 were promoted compared with the controls (Additional file 1: Figures S1, S2, S3, and S4, respectively). The biomass inoculated with E5 and S1 increased significantly compared with the controls (Fig. 5). Fresh and dry weights of roots and shoots of all the treatments were reduced by the increase of salt concentrations. However, the reduction of fresh and dry weights of shoots was much smaller for the treatments with the engineered strains (Fig. 5e, f). The engineered bacteria E5P and S1P promoted sprout growth more significantly than the wild strains E5 and S1, respectively (Fig. 5). Inoculation with E5 and E5P showed more beneficial effects on sprouts than that with S1 and S1P. Thus, the beneficial effects of endophytes on sprout growth were also influenced by bacterial taxa.

## Discussion

Ethylene is a gaseous plant growth hormone produced endogenously by almost all plants. Besides being a plant growth regulator, ethylene has also been established as a stress hormone [13]. Under a stress condition (e.g., salinity, drought, waterlogging, heavy metals, and pathogenicity), the endogenous production of ethylene is accelerated substantially, which adversely affects the root growth and consequently the plant growth as a whole [14]. Some soil microbes that produce the ACC deaminase promote plant growth by sequestering and cleaving plant produced ACC, and thereby reducing the plant ethylene level and allowing the plant to be more resistant to a wide variety of environmental stresses [10]. Genetically engineered plants expressing ACC deaminase activity are less subject to the deleterious effects of stress conditions on plant growth than non-transgenic plants [14, 15]. However, improvement of ACC deaminase traits in bacteria has not been well studied [14], probably attributable to that ACC deaminase substrate ACC is plant-produced and the ACC deaminase is localized within the cytoplasm of the bacteria, only the ACC taken up by the bacteria can be degraded [10]. In this study, the ACC deaminase was displayed on the endophytic *Enterobacteriaceae* strain cells, and the ACC excluded by plant tissues could contact directly with ACC deaminase and be degraded by engineered endophytes [16]. Thus the sprouts inoculated with the engineered strains showed higher ACC deaminase activities and lower ethylene contents than those with the wild endophytes under saline stress conditions.

Presently, a major constraint in engineered plant secondary metabolite production is that only a few regulatory genes are known [1], and results of over-expression of chosen enzymes are often disappointing due to lack of understanding of metabolic regulation [4]. Furthermore, plant metabolic engineering often requires transformation with multiple genes and most of the enzymes can be readily transformed into endophytic bacteria. Therefore, it is advantageous to use the surface display protocol of endophytic bacteria. In this study, the different wild and engineered endophytic bacteria showed different effects on the rice sprouts. Inoculation with E5 and E5P showed more beneficial effects on sprouts than that with S1 and S1P although the differences between ACC deaminase activities of E5 and S1 were not significant in vitro. The effects of endophytic bacteria on host plants may be influenced by their interactions with the host plants. Bacteria belonging to the genera *Enterobacter* are frequently associated with plants, colonizing in the rhizosphere and other plant parts [17]. The endophytic *Enterobacter* sp. has been found in multiple plant species and is used to prevent plant disease and to enhance phytoremediation [18, 19]. Through genome annotation and comparative genomics of *Enterobacter* sp. 638, a set of genes specific to the plant niche adaptation have been identified [20]. Therefore, we realize that expressing ACC deaminase gene on endophytic *Enterobacter* should improve saline tolerance of rice sprouts. The effects of engineered *Enterobacter* on other plants in stress conditions should be further studied [21].

In general, inoculation with engineered bacteria expressing ACC deaminases on bacterial cells can greatly alter ACC concentrations in plant tissues. The usage of engineered bacteria should be a very effective way to enhance plants growth under various stress conditions. The protocol of engineering bacterial endophytes shows great potential to over-express plant enzymes associated into complexes, which increases the efficiency of metabolic pathways by substrate exchange between enzymes.

## Conclusions

A protocol was developed to synthesize engineered strains by expressing ACC deaminase gene on the cells of endophytic *Enterobacter* sp. E5 and *Kosakonia* sp. S1. The engineered strains significantly increased ACC deaminase activities. Inoculation of the engineered strains increased deaminase activities of sprouts more than inoculation of the wild strains and the control, and reduced the ethylene concentration of sprouts. Inoculation of the engineered strains into rice seeds could improve saline resistance of seeds under salt concentrations from 10 to 25 g L^−1^ and promoted the growth of sprouts. The protocol of surface display systems of endophytic bacteria should be useful to utilize in engineering strains for plant secondary metabolite production.

## Methods

### Isolation of Enterobacteriaceae from rice sprouts

Rice (*Oryza sativa* L. cv. Wusimi) seeds were sterilized in the surface by sequential immersion in 75% (v/v) ethanol for 5 min and sodium hypochlorite solution (5% available chlorine) for 5 min. Then, the seeds were germinated on pieces of autoclaved filter paper soaked with sterile water without light at 25 °C. After 4 days, selected sprouts were excised aseptically for isolation of endophytic bacteria. The sprouts were cut into small pieces (about 0.2 × 0.4 cm), and put into selective Eosin-Methylene Blue (EMB) medium (Huankai, Guangzhou, China) with 1.5% agar for *Enterobacteriaceae* isolation [22]. Plates were incubated at 26 °C for 4 days for *Enterobacteriaceae* growth. The purified strains were further identified to the genus level with the 16S rDNA gene sequence analysis [23]. The sequence data have been deposited in the National Center for Biotechnology Information (NCBI) under the Accession Number KY800390 and KY8003901.

### Construction of surface display plasmids

The plasmids were constructed according to previous methods with some modifications [19, 24]. The whole gene contained promoter functional fragments without the 3′ end coding sequences (HQ834306, 565 bp), ice nucleation protein N terminate gene *inaK*-N (AF013159, 633 bp), and ACC deaminase gene *acdS* (JQ646055, 1017 bp) (Fig. 1b), which was synthesized by RealGene Bio. Corp. (Shanghai, China). The N-terminal domain of InaK acted as an anchoring motif for the display of ACC deaminase. Therefore, the bacterial transformants were expected to express, secrete, and display the ACC deaminase on the cell surface. The ligated fragments were digested with *Eco*RI and *Xba*I, and inserted into the corresponding site of plasmid pUC57. The recombinant plasmids were identified by double digestion and sequencing, and then transformed into endophytic bacterial cells with electroporator (BTX). The transformed clones were screened from the Luria–Bertani (LB) medium containing 300 μg mL^−1^ ampicillin. Twenty transformed clones were amplified for *inaK*-N gene. The selected clones were further inoculated into rice sprouts under different saline conditions.

### Quantification of ACC deaminase activity

The ACC deaminase activity was assayed by measuring the amount of α-ketobutyrate produced when the enzyme cleaved ACC [25]. The amount of α-ketobutyrate generated by the activity of ACC deaminase was calculated by subtracting the absorbance value of reagent mixture without ACC from the absorbance value of reagent and ACC.

### Quantification of ethylene in sprouts

To quantify the production of ethylene by the rice sprouts, rice seeds were grown in plates with 4 mL saline solutions of 10 g L^−1^ NaCl for 1 week. The wild and engineered strains were cultured for 24 h in LB and LB containing 100 μg mL^−1^ ampicillin, respectively. The bacterial cells were centrifuged at 4500*g* and resuspended in sterile water to an OD_600_ nm = 0.50. Rice seeds were inoculated with the strains and sterile water (the control). Ethylene concentrations in sprouts were determined with the ELISA Kits (Kmsbiotech, Shanghai, China) under the manufacturer’s instruction. The sensitivity of this assay was 1.0 pmol L^−1^.

### Saline stress of rice sprouts inoculated with engineered strains

The wild and engineered strains were cultured for 24 h in the LB medium without and with 100 μg mL^−1^ ampicillin, respectively. Bacterial cells were centrifuged at 4500*g* and resuspended in 10 mM MgCl_2_ to an OD_600_ nm = 0.50. Surface-sterilized seeds were inoculated with the bacterial suspension before seed germination. As the negative control, surface-sterilized seedlings were inoculated with 10 mM MgCl_2_. Saline solutions of 10, 15, 20, and 25 g L^−1^ NaCl (with electrical conductivities of 18.3, 25.2, 31.6, and 39.5 ms cm^−1^, respectively) were used to test saline tolerance of rice sprouts. Germination assays were conducted by placing 20 seeds in a 9 cm Petri dish on pieces of Whatman #4 paper moistened with sterile water and the saline solutions, respectively. Lengths, fresh weights, and dry weights of roots and shoots were measured. Measurements of the root and shoot parameters were conducted based on 100 seedlings for each treatment.

### Statistical analysis

Statistical analysis of data was carried out using the SPSS statistical package (version 16.0 for Windows, SPSS Inc.). For seed germination experiments, data were represented as mean ± standard deviation of five replicates. The data were subjected to analyses of variance (ANOVA) and treatment means were compared by Duncan’s multiple-range test. All analyses were performed at *P* < 0.05.

## Additional file

**Additional file 1.** Additional figures.
