# Supplementary material for: Surface display of ACC deaminase on endophytic Enterobacteriaceae strains to increase saline resistance of host rice sprouts by regulating plant ethylene synthesis
Source: Microb Cell Fact. 2017 Nov 28;16:214. doi: 10.1186/s12934-017-0831-5 (PMC5704625; doi:10.1186/s12934-017-0831-5)
Supplement: Supplementary file 1 — Additional file 1. Additional figures. [file 12934_2017_831_MOESM1_ESM.docx]

**Additional materials**

**Surface display of ACC deaminase on endophytic *Enterobacteriaceae* strains to increase saline resistance of host rice sprouts by regulating plant ethylene synthesis**

Authors:

Yupei Liu^1^, Lixiang Cao^2^*, Hongming Tan^2^, and Renduo Zhang^1^*

**Lixiang Cao** (Author for Correspondence)

Tel: 86- 20-84110238, fax: 86- 20-84036215, Email: [lssclx@163.com](mailto:lssclx@163.com)

**Renduo Zhang** (Author for Correspondence)

Tel: 86- 20-84110052, fax: 86- 20-84036215, E-mail: [zhangrd@mail.sysu.edu.cn](mailto:zhangrd@mail.sysu.edu.cn)

**S1 Results**

Seeds were treated with sterile water (the control), endophytic *Kosakonia* sp. S1 and its *acdS* gene surface expressed strain (i.e., the engineered strain S1P), endophytic *Enterobacter* sp. E5 and its engineered strain E5P. Germination of rice seeds during different periods under the saline stresses of 10, 15, 20, and 25 g L^-1^ is shown in Figs. S1, S2, S3, and S4, respectively.

**Figure legends**

Figure S1 Germination of rice seeds treated with sterile water (the control), endophytic *Kosakonia* sp. S1 and its *acdS* gene surface expressed strain (i.e., the engineered strain S1P), endophytic *Enterobacter* sp. E5 and its engineered strain E5P under the saline stress of 10 g L^-1^ during different periods.

Figure S2 Germination of rice seeds treated with sterile water (the control), endophytic *Kosakonia* sp. S1 and its *acdS* gene surface expressed strain (i.e., the engineered strain S1P), endophytic *Enterobacter* sp. E5 and its engineered strain E5P under the saline stress of 15 g L^-1^ during different periods.

Figure S3 Germination of rice seeds treated with sterile water (the control), endophytic *Kosakonia* sp. S1 and its *acdS* gene surface expressed strain (i.e., the engineered strain S1P), endophytic *Enterobacter* sp. E5 and its engineered strain E5P under the saline stress of 20 g L^-1^ during different periods.

Figure S4 Germination of rice seeds treated with sterile water (the control), endophytic *Kosakonia* sp. S1 and its *acdS* gene surface expressed strain (i.e., the engineered strain S1P), endophytic *Enterobacter* sp. E5 and its engineered strain E5P under the saline stress of 25 g L^-1^ during different periods.


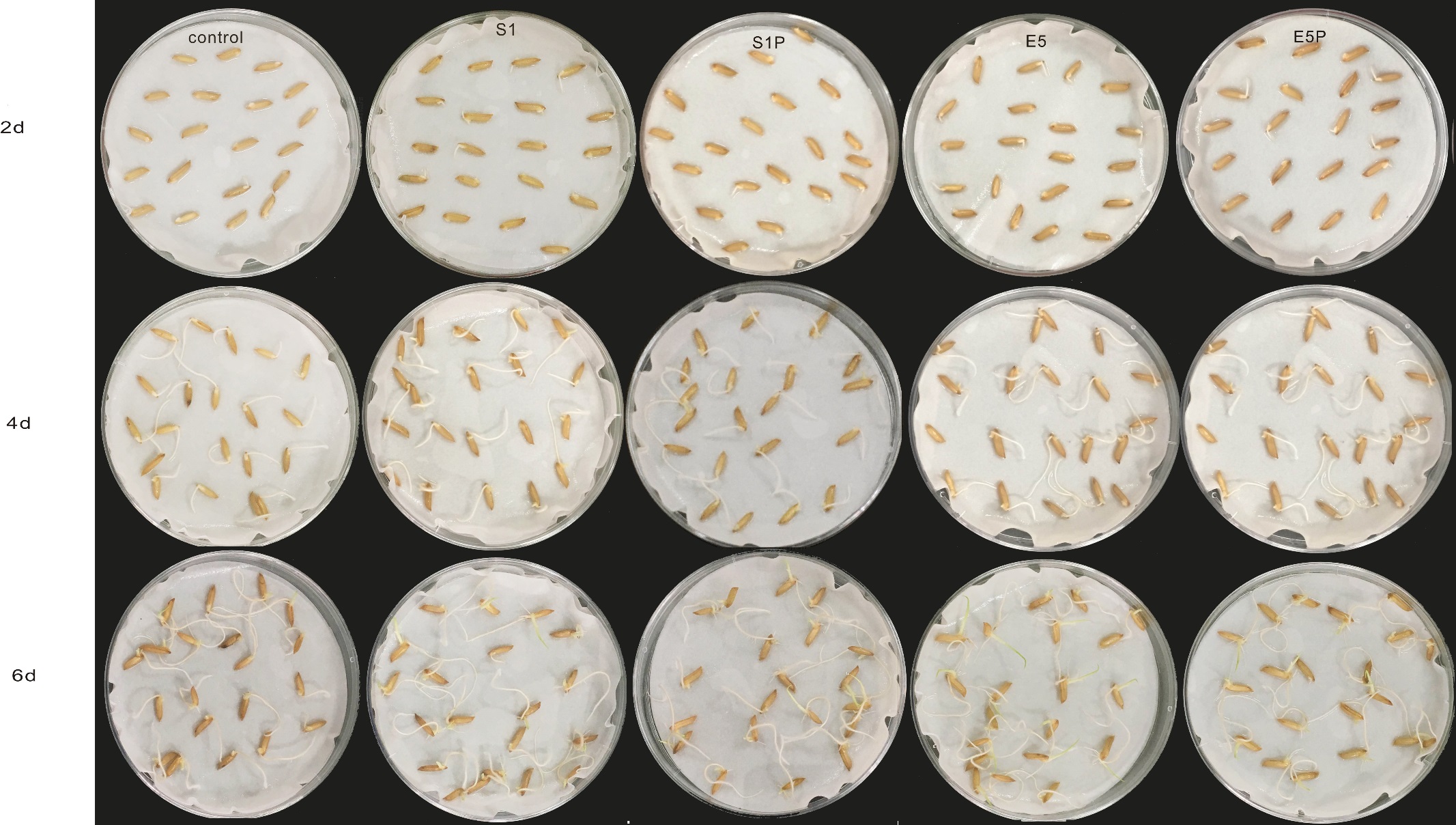


Figure S1


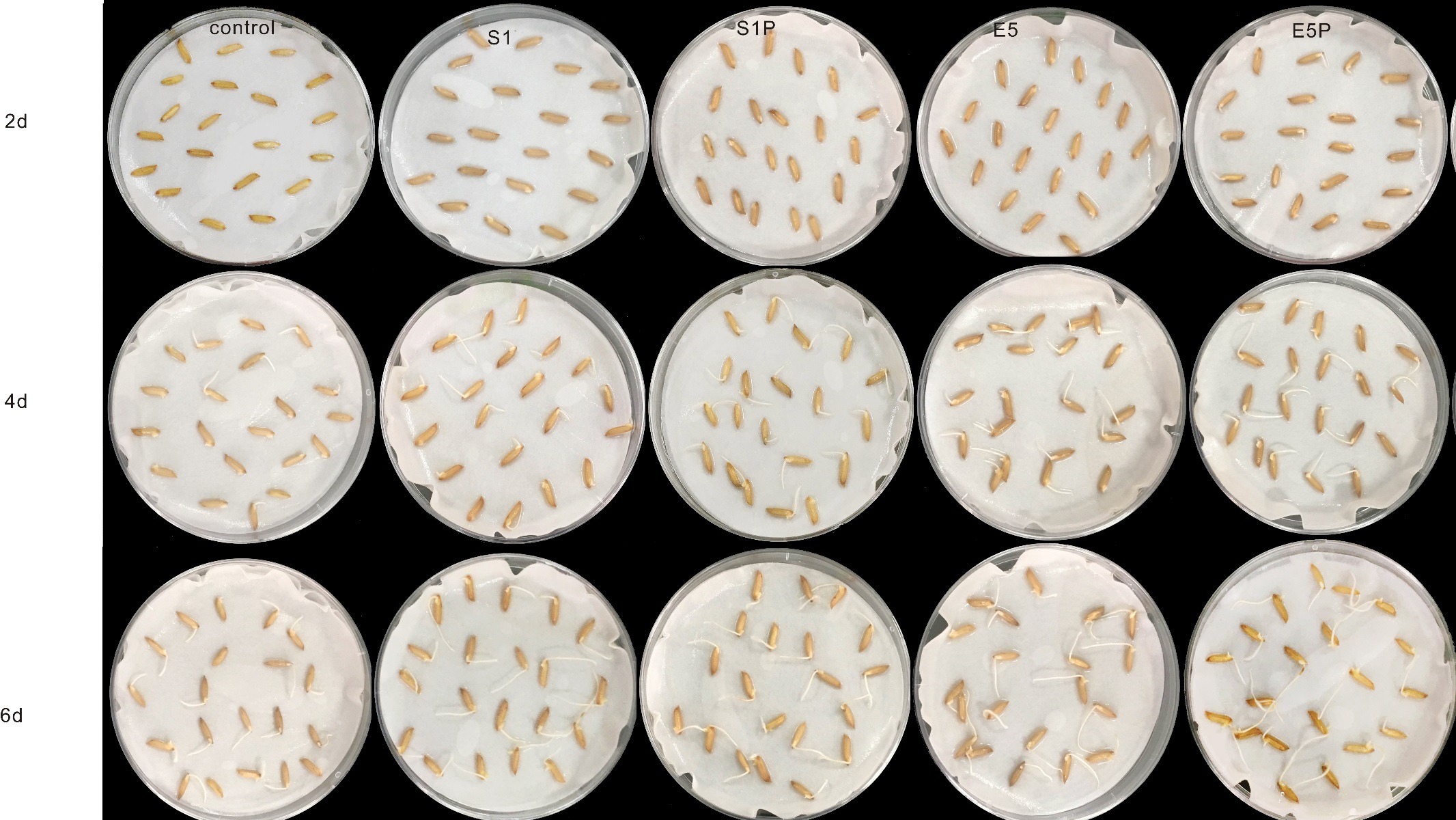


Figure S2


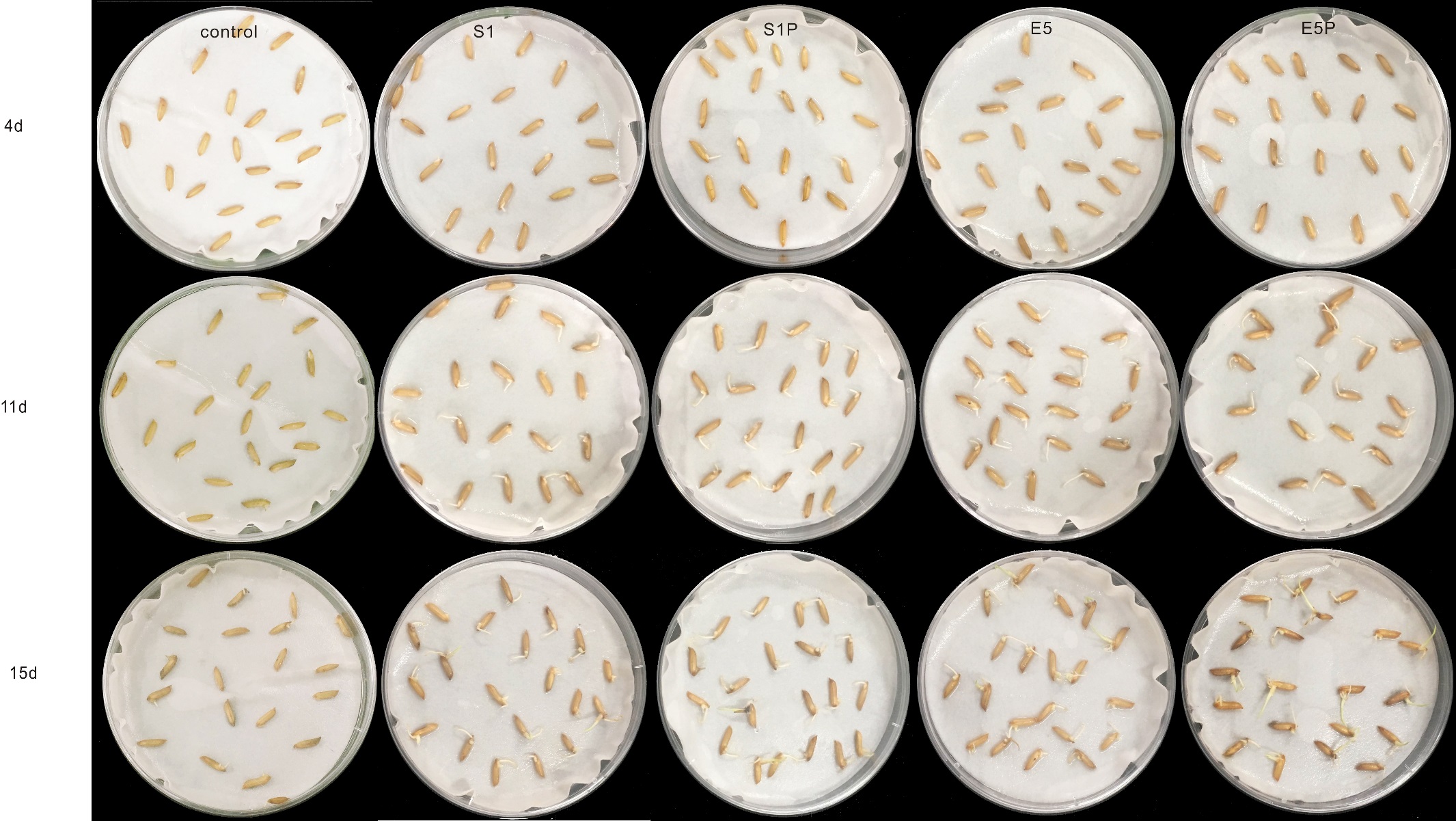


Figure S3


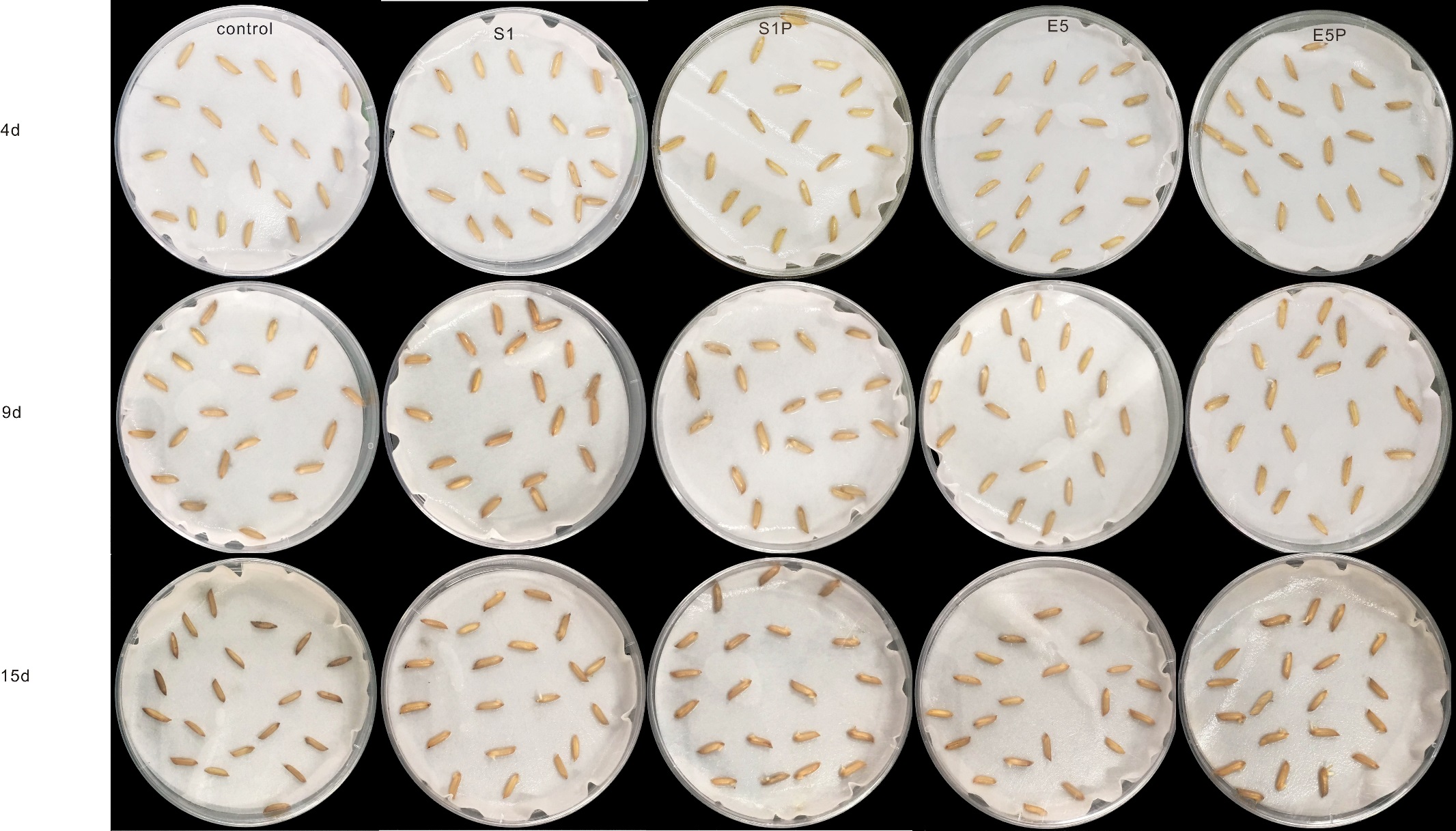


Figure S4
